# Protein fibril aggregation on red blood cells: a potential biomarker to distinguish neurodegenerative diseases from healthy aging

**DOI:** 10.1093/braincomms/fcae180

**Published:** 2024-06-13

**Authors:** Thomas Rudolf Schneider, Luisa Stöckli, Ansgar Felbecker, Peter Niraj Nirmalraj

**Affiliations:** Department of Neurology, Cantonal Hospital St. Gallen, St. Gallen CH-9007, Switzerland; Department of Neurology, Cantonal Hospital St. Gallen, St. Gallen CH-9007, Switzerland; Department of Neurology, Cantonal Hospital St. Gallen, St. Gallen CH-9007, Switzerland; Transport at Nanoscale Interfaces Laboratory, Swiss Federal Laboratories for Materials Science and Technology, Dübendorf CH-8600, Switzerland

**Keywords:** protein fibril aggregation, atomic force microscopy, red blood cells, aging, neurodegenerative diseases

## Abstract

Neurodegenerative diseases like Alzheimer’s disease are characterized by the accumulation of misfolded proteins into fibrils in the brain. Atomic force microscopy is a nanoscale imaging technique that can be used to resolve and quantify protein aggregates from oligomers to fibrils. Recently, we characterized protein fibrillar aggregates adsorbed on the surface of red blood cells with atomic force microscopy from patients with neurocognitive disorders, suggesting a novel Alzheimer’s disease biomarker. However, the age association of fibril deposits on red blood cells has not yet been studied in detail in healthy adults. Here, we used atomic force microscopy to visualize and quantify fibril coverage on red blood cells in 50 healthy adults and 37 memory clinic patients. Fibrillar protein deposits sporadically appeared in healthy individuals but were much more prevalent in patients with neurodegenerative disease, especially those with Alzheimer’s disease as confirmed by positive CSF amyloid beta 1–42/1–40 ratios. The prevalence of fibrils on the red blood cell surface did not significantly correlate with age in either healthy individuals or Alzheimer’s disease patients. The overlap in fibril prevalence on red blood cells between Alzheimer’s disease and amyloid-negative patients suggests that fibril deposition on red blood cells could occur in various neurodegenerative diseases. Quantifying red blood cell protein fibril morphology and prevalence on red blood cells could serve as a sensitive biomarker for neurodegeneration, distinguishing between healthy individuals and those with neurodegenerative diseases. Future studies that combine atomic force microscopy with immunofluorescence techniques in larger-scale studies could further identify the chemical nature of these fibrils, paving the way for a comprehensive, non-invasive biomarker platform for neurodegenerative diseases.

## Teaser

Schneider *et al*. employ atomic force microscopy to reveal that although fibril aggregation on red blood cells appears only sporadically in healthy individuals without age correlation, it significantly increases in neurodegenerative disease patients. This finding suggests its potential as an early marker to differentiate between normal aging and disease states.

## Graphical Abstract

**Graphical Abstract:**
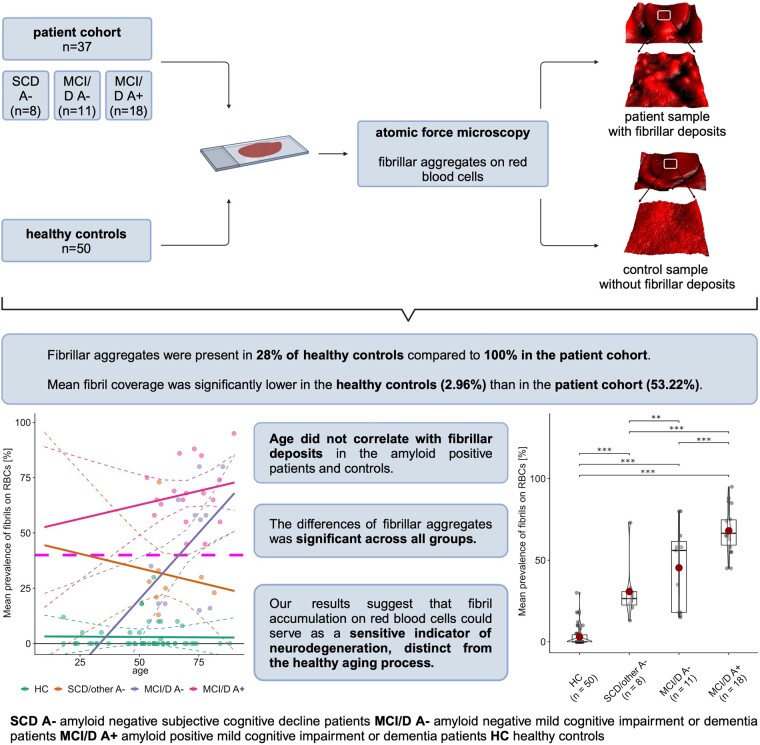

## Introduction

Neurodegenerative diseases are characterized by the progressive accumulation of misfolded proteins. Alzheimer’s disease, the most prevalent of these conditions, is responsible for ∼60–80% of all dementia cases.^1-3^ It is characterized by the formation of extracellular fibrillary β-amyloid (Aβ) brain plaques, which initiate a pathological cascade involving the intraneuronal aggregation and trans-synaptic spreading of fibrillary hyperphosphorylated tau proteins, neuroinflammation and oxidative stress, leading to neuronal injury and degeneration.^4,5^ Misfolded amyloid and tau proteins tend to aggregate into fibrils formed by amyloid beta-sheets that spread in a disease-specific pattern throughout the nervous system.^6-8^ Studies suggest that even minor reductions in Aβ and tau clearance, facilitated by the CSF’s bulk flow across the blood–brain barrier (BBB), perivascular circulation and glymphatic system, can disrupt the balance of Aβ production and clearance This imbalance triggers protein misfolding, aggregation and early extracellular build-up.^9-13^

Alzheimer’s disease pathology frequently co-occurs with α-synuclein, TDP-43 and vascular pathologies, highlighting the prevalence of multiple proteinopathies in neurodegenerative diseases.^14-16^ Although distinct proteins are involved in different neurodegenerative diseases, protein misfolding into fibrillary aggregates via oligomeric species formation is found to be a common mechanism shared by neurodegenerative diseases like Alzheimer’s disease,^17^ Parkinson’s disease and Lewy body disease,^18^ frontotemporal degeneration^19^ and amyotrophic lateral sclerosis.^20^

Aging significantly increases the risk of neurodegenerative diseases such as Alzheimer’s disease. With the incidence doubling roughly every 5 years post 65 years of age, the majority of cases are found in those 85 years and older.^21^ Therefore, exploring how aging influences protein aggregation in healthy individuals and neurodegenerative diseases is a question of significant importance, albeit currently understudied. Studies conducted on animal models suggest that protein aggregation is not confined to disease states but is a process associated with aging itself.^22,23^ However, certain proteins with a predisposition for aggregation might trigger the formation of disease-specific protein Aβ aggregates.^24^ Additionally, in vitro studies have demonstrated cross-seeding between Aβ and other aggregating proteins linked to neurodegenerative diseases, including α-synuclein, tau and TDP-43.^25-27^ Given that pathologic Aβ and tau proteins have been shown to adhere to surfaces,^28,29^ evaluating both soluble and insoluble protein aggregates, in CSF and blood could serve as potentially valuable biomarkers to distinguish between healthy aging and neurodegenerative diseases. Currently, only amyloid and tau PET imaging offers direct measurements of pathologic protein aggregation in Alzheimer’s disease.^30-32^ In contrast, soluble Aβ and tau species levels in CSF and plasma, while indicative of Alzheimer’s disease pathology, do not directly measure pathological protein misfolding and aggregation. In recent years, immuno-infrared sensors have shown reliable detection of misfolding and β-sheet structure formation of Aβ proteins in plasma of Alzheimer’s disease patients.^33,34^ This structural marker of Aβ misfolding can predict future clinical Alzheimer’s disease up to 17 years before clinical conversion.^35,36^

Resolving and measuring protein fibril deposits on red blood cells (RBCs), which have a lifespan of approximately 120 days (range 70–140 days),^37^ could provide valuable insights into protein misfolding and aggregation. Such a biomarker for neurodegenerative diseases could be more stable and less influenced by environmental and metabolic factors compared to plasma biomarkers,^38,39^ similar to how glycated haemoglobin^40^ is used in managing diabetes.^41^ Lan *et al*.^42^ demonstrated that Alzheimer’s disease patients exhibit significantly higher amyloid binding on RBCs and altered RBC morphology, using thioflavin T staining and immune fluorescence assays. Yet, standardized methods for detailed analysis and quantification of protein deposits on RBCs are lacking.

Atomic force microscopy (AFM) offers a promising approach to characterize protein aggregate morphology in blood samples in a label-free manner, providing insights into protein aggregation and neurodegenerative disease progression.^43-45^ AFM allows the study of protein aggregates from oligomers to fibrillar aggregates involved in Alzheimer’s disease pathogenesis, both in CSF^43,44,46^ and on the surface of RBCs, without the need for complex sample preparation steps. Preliminary data from our research indicate that fibrils are present in abundance on the surface of RBCs in patients with Alzheimer’s disease, suggesting that fibril coverage could potentially serve as a biomarker for Alzheimer’s disease pathology.^47^

Nevertheless, there is a clear need for more data on protein, particularly fibril aggregation, on RBCs in healthy aging individuals since we have previously only studied samples from a small group of blood donors (*n* = 8) lacking clinical characterization.^47^ This will enhance our understanding of fibril aggregates on RBCs as potential indicators of neurodegenerative diseases, particularly Alzheimer’s disease. To the best of our knowledge, only one recent study has reported an increase in hyper-stable protein aggregates in plasma with aging.^48^ However, this work did not investigate protein morphology or provide a clinical-level characterization of the plasma donors.

In this study, we use AFM to evaluate the burden of fibrils on RBCs in cognitively healthy adults spanning an age range from 18 to 88 years. We then compare this fibril coverage on RBCs between these healthy individuals and a heterogeneous memory clinic cohort previously published.^47^

## Materials and methods

### Study population

We analysed data from 50 cognitively healthy adults (HC) aged 18–88 years and 37 patients with cognitive complaints. Informed consent was obtained from all participants, and consent from accompanying relatives was obtained when necessary. The study procedures were approved by the ethics committee of East Switzerland (number: 202000558) and followed the Declaration of Helsinki.

### Cognitively unimpaired subjects

Between November 2021 and January 2022, we recruited 50 adult participants without cognitive complaints through media advertising. Participants underwent a general health questionnaire, the BrainCheck screening questionnaire for cognitive complaints,^49^ neurological examination and cognitive screenings [Montreal Cognitive Assessment (MoCA)^50^ and Quick Mild Cognitive Impairment (QMCI) Assessment^51^]. Participants with cognitive complaints, a MoCA score below 26/30 points or a QMCI score below 67/100 points, brain diseases and pathologic findings on clinical neurological examination were excluded.

### Patient cohort

We used data from the first 37 patients who presented with cognitive complaints at the memory clinic of the Department of Neurology at the Cantonal Hospital St. Gallen and underwent a lumbar puncture. The patient cohort was recruited between May and October 2020 and is described in our previous publication.^47^ Four patients were excluded from our previous analysis due to assumed implausible results with reduced Aβ42 and Aβ40 levels, but it is now recognized that reduced Aβ42 and Aβ40 levels can occur in neuroinflammatory conditions and CSF dynamics disorders.^52-55^ Therefore, data from all 37 patients with CSF were included in this analysis.

All patients underwent a comprehensive diagnostic workup comprising a neurological and neuropsychological examination, structural MRI imaging and CSF analyses. LUMIPULSE G assays (Fujirebio, Tokyo, Japan) were used to measure tTau Ag, pTau 181, β-amyloid 1–42, β-amyloid 1–40 and the corresponding Aβ 1–42/Aβ 1–40 ratio. Positive amyloid status was defined using standard laboratory cut-offs (CSF-β-amyloid 1–42/1–40 ratio < 0.068). Furthermore, the blood haemoglobin, creatinine and C-reactive protein levels as well as the CSF/serum quotient of albumin (*Q*_Alb_ = Alb_CSF_/Alb_Serum_ × 1000) as indicators of BBB function were recorded.

Clinical diagnoses according to diagnostic criteria were determined through interdisciplinary consensus. For further statistical analyses, patients were divided into three groups according to the cognitive status and evidence for neurodegeneration, as well as the amyloid status.

- SCD/other A−: eight patients with no evidence of neurodegenerative disease, including two with normal cognition, i.e. subjective cognitive disorder, and five with impaired cognition due to other causes (e.g. psychiatric disorder). All patients in this group tested negative for amyloid.
- MCI/D A−: eleven patients with cognitive impairments in either mild cognitive impairment (MCI) or dementia stage, showing evidence of a neurodegenerative disease who were amyloid-negative. Diagnoses included MCI due to vascular disease (MCI VD, *n* = 3), dementia due to vascular disease (VDD, *n* = 3),^56^ unspecified MCI (*n* = 1), idiopathic normal pressure hydrocephalus (*n* = 1),^57^ Parkinson’s disease dementia (*n* = 1) and Lewy body dementia (*n* = 1).^58^
- MCI/D A + (*n* = 18): eighteen patients with cognitive impairments in either MCI or dementia stage, showing evidence of amyloid positivity. The primary diagnoses were MCI due to Alzheimer’s disease (MCI AD, *n* = 2) or probable Alzheimer’s disease dementia with biomarker evidence (*n* = 14) according to the NIA-AA criteria of 2011.^59^ One patient was diagnosed with vascular dementia (dementia due to vascular disease), and another patient had a rapidly progressing dementia form (other).

### Sample handling and AFM measurements

**Figure 1:**
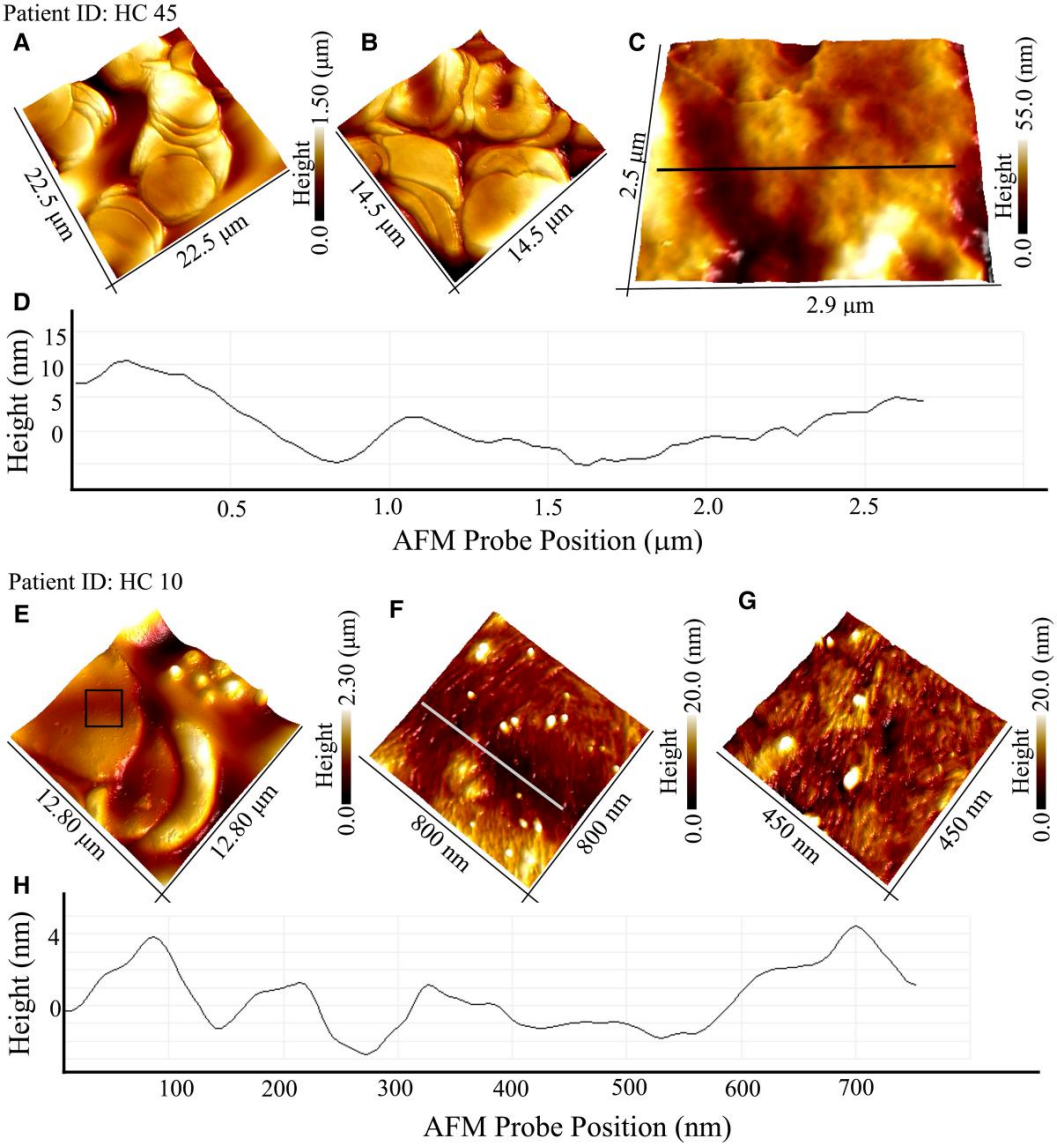
**Nanoscale imaging of protein fibrils on RBCs.** (**A**, **B**) Large-area and 3D-rendered AFM images of RBCs from HC (patient ID: 45). (**C**) Zoom-in AFM topography of RBC surface showing no evidence for fibrillar or even spherical particles on RBC membrane. The local differences in height on RBC stems from corrugations and lipid bilayer membrane damage. (**D**) Sectional profile extracted along the line indicated in AFM image shown in **C** revealing the local height differences across the RBC membrane. (**E**) 3D-rendered AFM image showing close-packed RBCs within a blood smear sample from a HC (ID: HC10). (**F**, **G**) Spatially magnified 3D AFM images showing the presence of fibrillar and spherical particles detected within the box region shown in **E**. (**H**) Sectional profile extracted along the line shown in **F**, revealing the local height differences across the RBC membrane. AFM, atomic force microscopy; RBC, red blood cell; HC, healthy control.

Six air-dried blood smears per participant were prepared within 24 h using about 50 μl of ethylenediaminetetraacetic acid blood each and stored at room temperature until transport into the laboratory facilities at Swiss Federal Laboratories for Materials Science and Technology, St. Gallen (EMPA). AFM measurements of blood smears are described in our previous study.^47^ In short, AFM measurements of blood smears were performed using Dimension Icon (Bruker) and SCOUT 150 HAR silicon AFM tips. AFM data were processed and analysed using Nanoscope analysis 1.9 (Bruker) software. Approximately 1000 RBCs on 30–50 scanned areas (50 µm²) recorded at different locations on the glass were analysed for each participant. The relative prevalence of protein fibrils on RBCs was calculated as the normalized surface coverage over an area of 5 µm² across the RBC membrane (see Fig. 1).

### Statistical analyses

Statistical analyses were performed in the R environment for statistical computing (version 4.3.0,^60^ R Project for Statistical Computing). The prevalence of fibrils on RBCs was recorded as zero (0%) if no fibrils were detected and served as the main outcome measure. Descriptive statistics, exploratory ANOVA tests and *χ*^2^ tests were conducted using the ‘arsenal’ package to compare clinical characteristics between disease groups^61^ (see Table 1). Linear regression models and ANOVA tests were used to explore the association between age and fibril aggregation on RBCs, including age, group and age × group interaction as predictors. Simple age regression slopes were analysed for each disease group separately using the ‘interaction’ package. Pairwise *t*-tests between groups were performed with Holm–Bonferroni adjustments for multiple testing using the ‘ggstats’ package.^62^ Additionally, receiver operating characteristic analyses were conducted using the ‘cutpointr’ package^63^ to differentiate between diagnosis groups based on the prevalence of fibrils on RBCs, optimizing the Youden index (sensitivity + specificity − 1). Exploratory linear regression analyses were conducted to investigate potential associations between haemoglobin levels, kidney function, systemic inflammation and BBB function in the patient group. Specifically, these analyses examined the relationships among haemoglobin, creatinine, C-reactive protein levels, albumin quotient (*Q*_Alb_) and the prevalence of fibrils on RBCs (see Supplementary Fig. 1).

**Table 1:** Clinical characteristics and AFM data of participants by diagnosis group

	HC (*N* = 50)	SCD/other A− (*N* = 8)	MCI/D A− (*N* = 11)	MCI/D A+ (*N* = 18)	Total (*N* = 87)	*P*-value
Gender						<0.001^a^
Male	8 (16.0%)	6 (75.0%)	7 (63.6%)	5 (27.8%)	26 (29.9%)	
Female	42 (84.0%)	2 (25.0%)	4 (36.4%)	13 (72.2%)	61 (70.1%)	
Age						<0.001^b^
Mean (SD)	51.56 (16.67)	63.02 (10.02)	71.12 (9.70)	70.94 (10.36)	59.10 (16.76)	
Range	18.00–88.00	54.10–81.50	54.90–85.10	53.20–89.80	18.00–89.80	
Years of education	14.72 (2.72)	13.86 (4.02)	11.70 (3.97)	11.65 (2.76)	13.67 (3.27)	<0.001^b^
Adjusted MoCA score (/30)	29.14 (1.03)	25.29 (2.14)	19.20 (3.33)	16.18 (5.36)	25.01 (6.17)	<0.001^b^
QMCI	84.09 (6.13)	NA	NA	NA	84.09 (6.13)	
CSF amyloid 1–42/1–40 ratio	NA	0.09 (0.01)	0.10 (0.01)	0.04 (0.01)	0.07 (0.03)	<0.001^b^
CSF p-tau (ng**/**l)	NA	30.81 (9.07)	35.43 (14.40)	115.23 (47.59)	73.25 (53.49)	<0.001^b^
CSF t-tau (ng**/**l)	NA	219.38 (71.95)	254.00 (116.23)	716.67 (301.46)	477.64 (327.98)	<0.001^b^
Mean prevalence of fibrils on RBCs (%)						<0.001^b^
Mean (SD)	2.96 (6.10)	30.75 (18.13)	45.36 (25.70)	68.00 (14.09)	24.33 (29.82)	
Mean (confidence interval)	2.96 (1.23, 4.69)	30.75 (15.59, 45.91)	45.36 (28.10, 62.63)	68.00 (60.99, 75.01)	24.33 (17.98, 30.69)	
Range	0.00–30.00	13.00–73.00	15.00–80.00	45.00–95.00	0.00–95.00	

The mean and standard deviation (in parentheses), as well as range and 95% confidence interval of selected variables, are given. *P*-values of between-group differences are based on ANOVA tests for numerical data and on *χ*^2^ tests for categorical data. NA, not available.

^a^Pearson’s *χ*^2^ test.

^b^Linear model ANOVA.

## Results

### Clinical characteristics

Table 1 presents group summary statistics of clinical characteristics, CSF tTau Ag, pTau 181 and the Aβ 1–42/Aβ 1–40 ratio, the MoCA and QMCI results and the prevalence of fibrils on RBCs (for individual data, see Supplementary Tables 1 and 2). The HC group had a mean age of 51.6 years (SD = 16.7) within the age range of 18 to 88 years which was lower than in the patient groups. The majority of participants in both control and patient groups were female (70%). As expected, MoCA test results differed significantly among groups, with the HC group showing the highest scores. In the HC group, all participants achieved a MoCA score of 26 or higher (29.1 ± 1.0), and their QMCI scores ranged from 68.5 to 93 points (84.1 ± 6.13%). Clinical neurological examinations were unremarkable in all HC participants. Among the HC participants, 21 out of 50 reported a first-degree family member with dementia syndrome.

### Qualitative findings of AFM analysis of RBCs from healthy controls

Previously, we reported on AFM-based analysis of RBCs from a mixed cohort of 50 memory clinic patients.^47^ The differences in protein aggregate morphology from annular oligomers and protofibrils to mature fibrils were resolved and quantified. In the present study, we map the morphology of RBCs from 50 HC participants using AFM adopting the same methodology as previously desribed.^47^ We present detailed AFM morphology analysis for two study participants (HC-45 and HC-10) as they represent the structural diversity of all the RBCs analysed in blood smears from all 50 HC participants. Figure 1A and B are large-area AFM images rendered three-dimensionally (3D) showing RBCs recorded at different locations within a blood smear sample from HC-45. The surface topography was relatively featureless, as seen from the AFM image (Fig. 1C) and cross-sectional profile (Fig. 1D) when compared to RBCs from Alzheimer’s disease patients,^47^ which was observed to be highly corrugated with protein aggregate deposits. Next, we present results as an example from a participant whose RBCs were observed to contain fibrillar aggregates. Figure 1E is an AFM topography of close-packed RBCs present in blood smears from HC-10. Spatially magnifying within the region indicated by the box in Fig. 1E reveals the presence of both fibrillar and spherical aggregates as shown in 3D-rendered AFM images (Fig. 1F and G) and from cross-sectional profile (Fig. 1H). The length, size and prevalence of the fibrils across the RBCs are then quantified from the respective AFM topography.

### Prevalence of fibrils on RBCs in HC and A+ patients in relation to age and laboratory parameters

**Figure 2:**
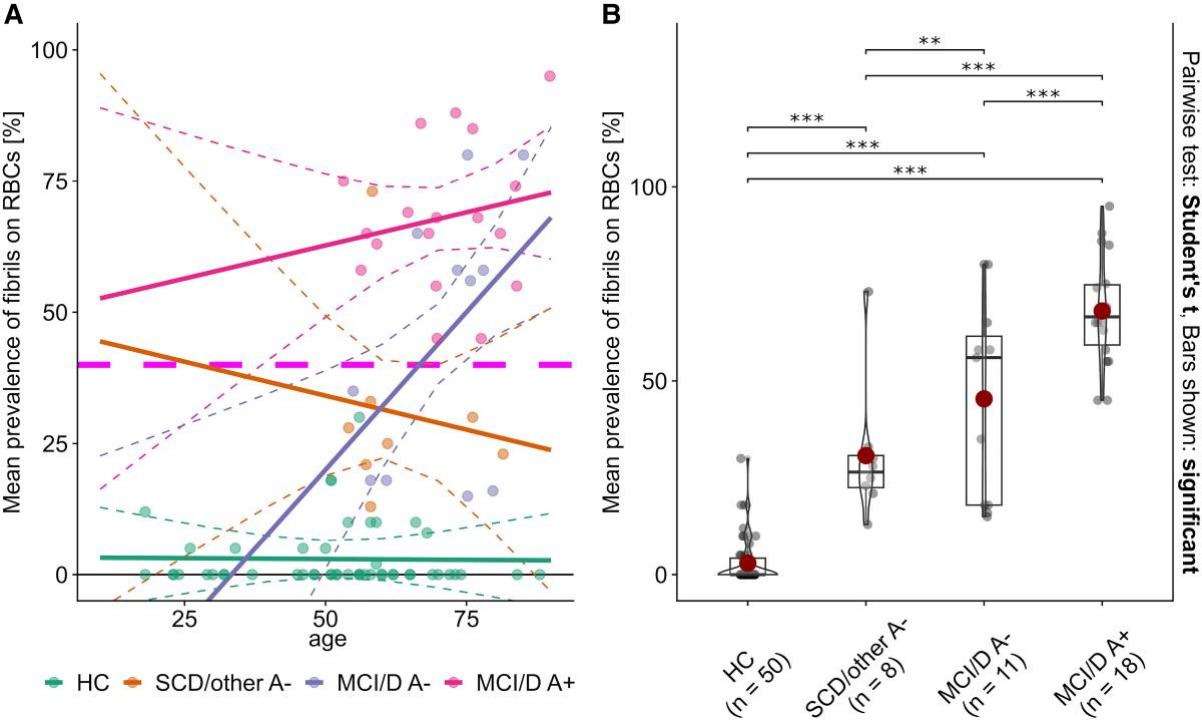
**Prevalence of fibrils on RBCs across age and groups.** (**A**) Prevalence of fibrils on RBCs plotted against age for each group, with regression lines and 95% confidence intervals indicating the simple slopes of age within each group. A horizontal dotted line, marking a 40% prevalence of fibrils on RBCs, represents the previously established cut-off for indicating amyloid positivity in CSF.^47^ A significant age-related increase in fibril prevalence was observed only in the ‘MCI/D A−’ group [slope 1.2%/year, 95% confidence interval (0.4, 2.0), *P* = 0.005]. No significant correlations were found in the HC (*P* = 0.95), ‘SCD/other A−’ (*P* = 0.59) or ‘MCI/D A+’ (*P* = 0.40) groups. (**B**) Box and whisker plots combined with violin plots in the Tukey style, representing the mean fibril prevalence on RBCs for each group. Significant pairwise differences between groups after Holm adjustment are noted above the brackets (*P* < 0.05, **P < 0.01, and ***P < 0.001). Results indicate significant differences between HC and both MCI/D A+ (*P* < 0.001) and MCI/D A− (*P* < 0.001) and between SCD/other A− and both MCI groups (A+ *P* < 0.001; A− *P* = 0.018). The comparison between the MCI/D A+ and MCI/D A− groups also shows a significant difference (*P* < 0.001). RBC, red blood cell; HC, healthy control; MCI/D A−, mild cognitive impairment/dementia, amyloid-negative; SCD/other A−, subjective cognitive decline/other, amyloid-negative); MCI/D A+, mild cognitive impairment/dementia, amyloid-positive.

In the multiple linear regression model, we observed a significant main effect of group (*F* = 2.9, *P* = 0.04) but not of age (*F* = 2.8, *P* = 0.096). There was a significant interaction between age and group (*F* = 3.0, *P* = 0.04). Simple slope analyses revealed a significant correlation between age and fibril prevalence only in the ‘MCI/D A−’ group (*P* < 0.01), while no significant associations were found in the HC (*P* = 0.95), ‘SCD/other A−’ (*P* = 0.59) or ‘MCI/D A+’ (*P* = 0.40) groups (see Fig. 2A). No significant correlations were found in the patient group between haemoglobin, creatinine, C-reactive protein levels, albumin quotient (*Q*_Alb_), and the prevalence of fibrils on RBCs (see Supplementary Fig. 1).

### Distinct group differences in the prevalence of fibrils on RBCs

Fibrillary protein aggregates on RBCs were detected in 14 out of 50 HC participants and in all patients. The measured prevalence of fibrils on RBCs ranged from 0 to ∼30% surface coverage. Among the 14 HC participants exhibiting positive fibril detection, 4 reported a first-degree family history of dementia. Notably, these subjects did not present with the highest fibril levels, recording values at 2, 5, 8, and 10%. Conversely, seven HC subjects showed fibril levels above 10% but lacked a family history of dementia.

Significant group differences were found in the mean prevalence of fibrils on RBCs (see Fig. 2B). The HC subjects had a significantly lower prevalence (2.96 ± 6.1%) compared to all three patient groups (*P* < 0.001). Furthermore, the ‘SCD/other A−’ group had a lower prevalence (30.8 ± 18.1%) compared to both the ‘MCI/D A−’ group (45.4 ± 25.7%, *P* = 0.02) and the ‘MCI/D A+’ group (68.0 ± 14.1, *P* < 0.001). Lastly, the ‘MCI/D A+’ group showed a significantly higher fibril prevalence than the ‘MCI/D A−’ group (*P* < 0.001).

### Exploratory receiver operating characteristic analyses

**Figure 3:**
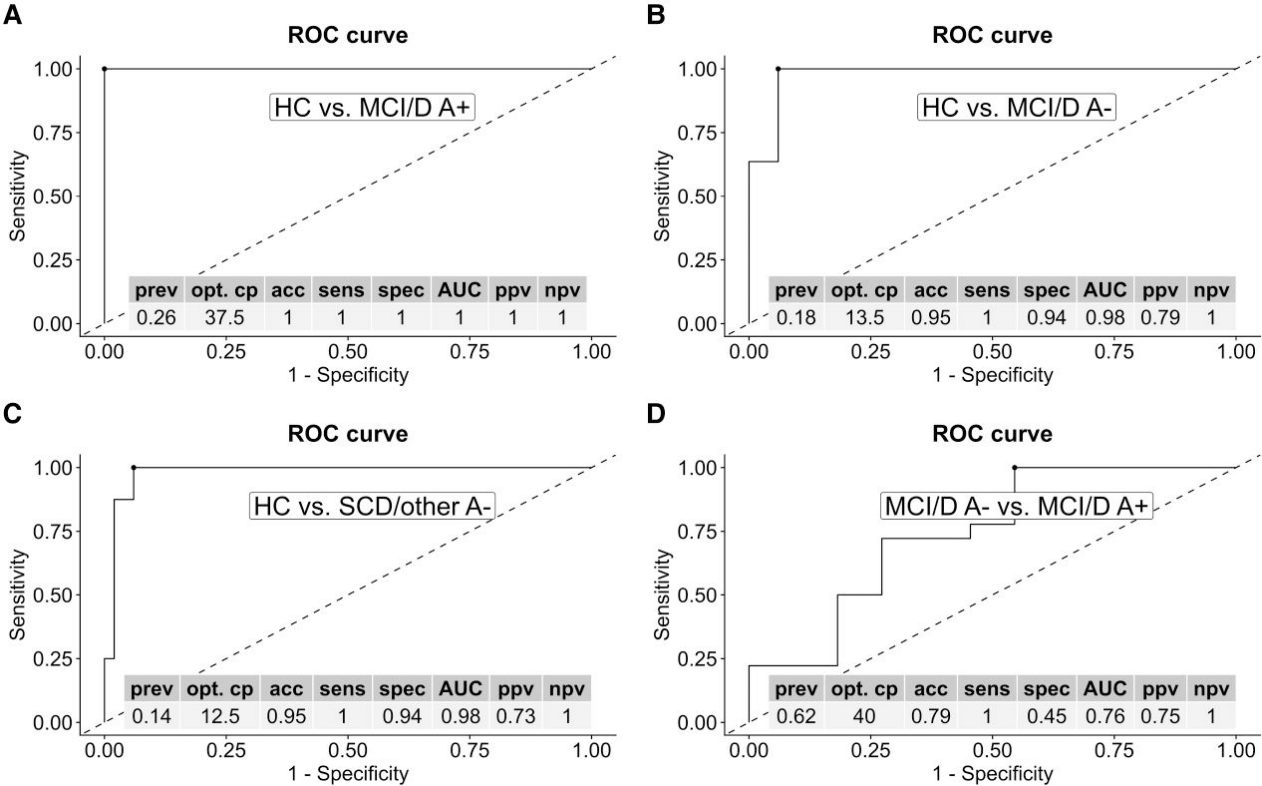
**Receiver operating characteristic analyses and diagnostic performance measures of the prevalence of fibrils on RBCs for differentiating between participants in the HC group and the patient groups (A–C), as well as between patients with cognitive impairments due to neurodegenerative diseases who are amyloid-positive (MCI/D A+) and those who are amyloid-negative (MCI/D A−) (D).** RBC, red blood cell; HC, healthy control; MCI/D A−, mild cognitive impairment/dementia, amyloid-negative; SCD/other A−, subjective cognitive decline/other, amyloid-negative; MCI/D A+, mild cognitive impairment/dementia, amyloid-positive; prev, prevalence of disease; opt. cp, optimal cut point determined by the Youden index; acc, accuracy; sens, sensitivity; spec, specificity; AUC, area under the curve; ppv, positive predictive value; npv, negative predictive value.

Given the distinct difference in fibril prevalence between the HC group and the other groups, various cut-offs (ranging from 12.5 to 37.5%) accurately differentiated the HC group from the others (accuracy 0.95–1.0, area under the curve 0.98–1; see Fig. 3A–C). In contrast, the fibril prevalence between the ‘MCI/D A+’ and ‘MCI/D A−’ groups overlapped, resulting in a moderate accuracy (0.79) and area under the curve (0.76) when using a fibril prevalence cut-off of 40% to differentiate between these groups (see Fig. 3D).

## Discussion

Our study investigates fibril aggregation on RBCs across healthy aging and its implications as biomarker for neurodegenerative diseases, particularly Alzheimer’s disease. Employing AFM, we quantified the extent of fibril aggregates on RBCs in blood smear samples from healthy adults and compared these measurements with those from a heterogeneous memory clinic cohort.^47^ The results provide preliminary insights into the occurrence and extent of fibril aggregation on RBCs in healthy aging, suggesting a potential role as a biomarker of neurodegenerative diseases.

Fibrillar deposits on RBCs were sporadically detected in 14 out of 50 healthy aging individuals, and when present, they displayed a lower prevalence on RBCs compared to patients with neurodegenerative diseases and also memory clinic patients with no clinical evidence of neurodegenerative disease. Notably, patients with Alzheimer’s disease pathology, confirmed by a positive CSF Aβ 1–42/1–40 ratio, showed the highest levels of fibrils on RBCs, suggesting a correlation with Alzheimer’s disease pathology. However, there was a significant overlap in the prevalence of fibrillar aggregates on RBCs between amyloid-positive and amyloid-negative patients but not with healthy controls (HC). Interestingly, we found no substantial correlation between age and fibril prevalence among cognitively healthy participants, patients with no evidence of neurodegeneration or amyloid-positive patients. Nonetheless, age-related increases were evident in a small, heterogeneous group of amyloid-negative patients with non-Alzheimer’s disease neurodegenerative/vascular diseases, necessitating further exploration in larger cohorts.

Preliminary receiver operating characteristic analyses demonstrated that different cut-offs of fibril prevalence accurately differentiated cognitively healthy individuals from patient groups. However, the overlap in fibril prevalence posed a limitation when distinguishing between amyloid-positive and amyloid-negative patients with neurodegenerative disease, suggesting that RBC fibril deposition may not be exclusive to Alzheimer’s disease but might also be present in other neurodegenerative diseases. We also noted significant fibril coverage on RBCs in amyloid-negative patients with vascular cognitive disorders, suggesting that these fibrils may derive from proteins associated with non-Alzheimer’s disease neurodegenerative diseases, considering the frequent overlap of neurodegenerative pathologies in dementia patients.^14,64^ We discovered a notable prevalence of fibril aggregates on RBCs in neurodegenerative disease patients, particularly in Alzheimer’s disease cases. This supports previous research indicating increased Aβ binding in Alzheimer’s disease patient RBCs and aligns with immuno-infrared-sensor studies linking Aβ misfolding in plasma to brain amyloidosis and early Alzheimer’s disease risk in the elderly.^33-36,42^ This could indicate that the pathological process of protein misfolding and aggregation in the brain involved in neurodegenerative diseases like Alzheimer’s disease,^17^ Parkinson’s disease and Lewy body disease,^18^ frontotemporal degeneration^19^ and amyotrophic lateral sclerosis^20^ may be detected in peripheral blood samples.

Recently, we detailed the protein morphology on RBCs within the analysed memory clinic cohort^47^ noting that among annular, spherical and fibrillar aggregates, only fibrils were consistently observed across all patients, with distinct morphological variations correlating with cognitive decline stages. RBCs of patients with mild Alzheimer’s disease were characterized by nodular, single-strand fibrils, suggesting protofibrillar aggregates, whereas RBCs from patients with severe Alzheimer’s disease–featured complex, intertwined, double-strand fibrils, indicative of mature fibrils. In the current study, RBCs from HC exhibited short, thin fibrils with protofibril morphology. These observations suggest fibril growth and evolution from oligomers to mature fibrils on RBCs, as the passage of mature, double-stranded fibrils from the brain across the BBB is improbable. The likelihood of oligomers adhering to RBCs, considering the Aβ42 peptide’s inherent tendency to aggregate, supports this hypothesis.^29,65^ An advantage of measuring protein aggregates on RBCs instead of soluble oligomers in plasma is the longevity of RBCs of approximately 120 days on average which may render measures of fibrils on RBCs more robust to variation environmental factors and the kidney function. However, interindividual differences in the life span of RBCs^37^ might lead to variation in fibril levels as has been observed in studies of glycated haemoglobin in diabetes management .^66^ BBB dysfunction, known to occur in Alzheimer’s disease patients,^67,68^ may facilitate the release of Aβ42 monomers into the bloodstream^69^ and could therefore influence the fibril prevalence on RBCs. However, in our pilot memory clinic cohort, only one Alzheimer’s disease dementia patient exhibited a CSF/serum albumin ratio indicative of BBB dysfunction, and we observed no correlation between BBB integrity and fibril prevalence.

### Limitations and future directions

Our findings are preliminary due to several important limitations, notably the lack of chemical characterization of the fibrils.^46,47^ Without this chemical information, no definitive conclusions about the cerebral origin of the fibrils can be drawn. Moreover, the reasons behind the increased fibril presence on RBCs in neurodegenerative diseases—whether a result of enhanced seeding and formation of fibrils by disease-specific proteins or impaired clearance from the bloodstream—remain to be determined. Extensive research has shown that specific protein seeds can form varying structures depending on their environment, as evidenced by studies on tau fragments isolated from Alzheimer’s disease brain tissues.^70-72^ In line with this, we have recently demonstrated that ultra-long fibrils, extending into the micrometre range, are present in the CSF of Alzheimer’s disease patients, differing significantly from the fibrils in the nanometre range observed on RBCs.^46^

The second major limitation is the small sample size of the patient cohort which is due to the labour intensity of AFM and limited resources. Furthermore, our study did not include the evaluation of CSF Alzheimer’s disease biomarkers in the HC group or conduct standard laboratory panels.

The principal challenges in using AFM techniques for studying proteins in neurodegenerative disease as potential biomarker candidates include integrating protein identification methods with AFM and automating the analysis. Automating the AFM for high image capture and machine learning guided data analysis could address throughput challenges. To improve fibril detection and characterization specificity, future research should consider combining AFM with techniques like fluorescent microscopy, which tags protein aggregates with specific fluorophores.^73^ Incorporating AFM with immunofluorescence–AFM techniques that identify Aβ, tau, TDP-43 and α-synuclein peptides in detailed protein structures would enable thorough analysis of protein aggregation in body fluids. This approach could offer a biomarker platform for detecting the presence and stage of prevalent neurodegenerative diseases using just a blood or CSF sample, fulfilling the unmet need for non-invasive biomarkers, particularly for non-Alzheimer’s disease, neurodegenerative diseases.

## Conclusion

Despite the highlighted limitations, our preliminary results provide novel insights into the occurrence and extent of fibril aggregation on RBCs in healthy aging and neurodegenerative diseases. Our results suggest that fibril accumulation on RBCs could serve as a sensitive indicator of neurodegeneration, distinct from the healthy aging process, potentially offering a diagnostic means to differentiate between healthy individuals and those with neurodegenerative disease, particularly Alzheimer’s disease. Notably, the observed increase in fibril deposition on RBCs among patients with subjective cognitive decline or cognitive impairments, in the absence of confirmed neurodegeneration, points to the possibility of undiagnosed neurodegenerative diseases within this group. In addition, AFM measurements on RBCs prepared from whole blood collected at different time points from the same patient will also help clarify the role of RBC’s age-dependent accumulation of protein aggregates. Future research that combines AFM with immunofluorescence could not only enhance our understanding of protein aggregation on RBCs but also aid in identifying specific neurodegenerative diseases using blood samples. The necessity for larger cohort studies employing advanced immunofluorescence–AFM techniques is evident to verify these initial findings and to validate the potential diagnostic value of RBC fibril analysis in neurodegenerative diseases.

## Supplementary Material

fcae180_Supplementary_Data

## Data Availability

All data needed to evaluate the conclusions in the paper are presented in the paper. Additional data related to this paper may be requested from the authors.
