# Supplementary material for: Protein fibril aggregation on red blood cells: a potential biomarker to distinguish neurodegenerative diseases from healthy aging
Source: Brain Commun. 2024 Jun 13;6(3):fcae180. doi: 10.1093/braincomms/fcae180 (PMC11170662; doi:10.1093/braincomms/fcae180)
Supplement: fcae180_Supplementary_Data [file fcae180_supplementary_data.pdf]

# SUPPLEMENTARY MATERIAL

## Protein fibril aggregation on red blood cells: a potential biomarker to distinguish neurodegenerative diseases from healthy aging

## Table of contents

**Supplementary Table 1:** Clinical characteristics and the fibril prevalence on RBCs of healthy controls. .....2
**Supplementary Table 2:** Clinical characteristics and the fibril prevalence on RBCs of the memory clinic patients cohort. ....................................................................................................................................3
**Supplementary Figure 1**: Correlations between laboratory parameters and fibril prevalence on RBCs.......4

**Supplementary Table 1:** Clinical characteristics and the fibril prevalence on RBCs of healthy controls.

| ID | Age | Group | MoCA score [0-30] | Prevalence of fibrillar aggregates on RBCs [%] |
|---|---|---|---|---|
| C-1 | 37 | HC | 27,5 | 0 |
| C-2 | 48 | HC | 29 | 0 |
| C-3 | 56 | HC | 30 | 30 |
| C-4 | 74 | HC | 30 | 0 |
| C-5 | 54 | HC | 29 | 0 |
| C-6 | 54 | HC | 29 | 10 |
| C-7 | 26 | HC | 29 | 5 |
| C-8 | 24 | HC | 28 | 0 |
| C-9 | 56 | HC | 28 | 0 |
| C-10 | 51 | HC | 30 | 18 |
| C-11 | 29 | HC | 29 | 0 |
| C-12 | 32 | HC | 30 | 0 |
| C-13 | 18 | HC | 30 | 12 |
| C-14 | 30 | HC | 30 | 0 |
| C-15 | 32 | HC | 29 | 0 |
| C-16 | 51 | HC | 30 | 18 |
| C-17 | 60 | HC | 28 | 0 |
| C-18 | 46 | HC | 27 | 0 |
| C-19 | 68 | HC | 29 | 8 |
| C-20 | 56 | HC | 29 | 0 |
| C-21 | 52 | HC | 29 | 0 |
| C-22 | 34 | HC | 30 | 5 |
| C-23 | 51 | HC | 29 | 0 |
| C-24 | 50 | HC | 28 | 5 |
| C-25 | 48 | HC | 30 | 0 |
| C-26 | 23 | HC | 30 | 0 |
| C-27 | 60 | HC | 30 | 0 |
| C-28 | 70 | HC | 27 | 0 |
| C-29 | 65 | HC | 30 | 0 |
| C-30 | 23 | HC | 30 | 0 |
| C-31 | 57 | HC | 30 | 0 |
| C-32 | 73 | HC | 30 | 0 |
| C-33 | 58 | HC | 28 | 10 |
| C-34 | 84 | HC | 29,5 | 0 |
| C-35 | 88 | HC | 29 | 0 |
| C-36 | 62 | HC | 26 | 0 |
| C-37 | 56 | HC | 30 | 0 |
| C-38 | 59 | HC | 30 | 10 |
| C-39 | 51 | HC | 28 | 0 |
| C-40 | 45 | HC | 28 | 0 |
| C-41 | 65 | HC | 28 | 0 |
| C-42 | 59 | HC | 30 | 2 |
| C-43 | 62 | HC | 30 | 0 |
| C-44 | 46 | HC | 28 | 5 |
| C-45 | 52 | HC | 30 | 0 |
| C-46 | 18 | HC | 30 | 0 |
| C-47 | 72 | HC | 30 | 0 |
| C-48 | 66 | HC | 29 | 10 |
| C-49 | 58 | HC | 30 | 0 |
| C-50 | 69 | HC | 30 | 0 |

**Supplementary Table 2:** Clinical characteristics and the fibril prevalence on RBCs of the memory clinic patients cohort.

| ID | Age | Group | Main Dx | Secondary Dx | Dx specifier | MoCA score [0-30] | Aβ42/Aβ40 ratio | p-Tau [ng/l] | t-Tau [ng/l] | Prevalence of fibrillar aggregates on RBCs [%] |
|---|---|---|---|---|---|---|---|---|---|---|
| **KSSG-8** | 61 | SCD/other A- | Other | | Occlusive Hydrocephalus | 24 | 0,084 | 34 | 208 | 25 |
| **KSSG-17** | 58 | SCD/other A- | Other | | Cognitive disorder due to psychiatric disorder | 22 | 0,097 | 23 | 148 | 13 |
| **KSSG-19** | 58 | SCD/other A- | SCD | | | 27 | 0,089 | 25 | 146 | 33 |
| **KSSG-24** | 54 | SCD/other A- | Other | | Cognitive disorder due to psychiatric disorder | 24 | 0,099 | 38 | 299 | 28 |
| **KSSG-28** | 57 | SCD/other A- | Other | | Cognitive disorder due to psychiatric disorder | 27 | 0,073 | 31 | 213 | 21 |
| **KSSG-30** | 82 | SCD/other A- | Left parietooccipital atrophy of unknown etiology | | | 28 | 0,081 | 42 | 272 | 23 |
| **KSSG-46** | 76 | SCD/other A- | SCD | | | 25 | 0,073 | 37 | 324 | 30 |
| **KSSG-49** | 58 | SCD/other A- | Other | | Cognitive disorder due to psychiatric disorder | | 0,092 | 16 | 145 | 73 |
| **KSSG-10** | 66 | MCI/D A- | VDD | | | 16 | 0,104 | 26 | 161 | 65 |
| **KSSG-20** | 75 | MCI/D A- | PDD | | | 20 | 0,094 | 51 | 357 | 15 |
| **KSSG-22** | 85 | MCI/D A- | MCI VD | | | 23 | 0,077 | 36 | 264 | 80 |
| **KSSG-27** | 75 | MCI/D A- | VDD | | | 18 | 0,089 | 17 | 146 | 80 |
| **KSSG-29** | 55 | MCI/D A- | MCI VD | | | 25 | 0,098 | 35 | 226 | 35 |
| **KSSG-35** | 76 | MCI/D A- | MCI unsp. | | | 22 | 0,103 | 30 | 171 | 56 |
| **KSSG-37** | 61 | MCI/D A- | MCI unsp. | | Alcohol dependence | 20 | 0,1 | 28 | 189 | 18 |
| **KSSG-41** | 58 | MCI/D A- | MCI VD | | | 16 | 0,114 | 46 | 254 | 18 |
| **KSSG-42** | 80 | MCI/D A- | Possible NPH | | | 16 | 0,097 | 34 | 238 | 16 |
| **KSSG-44** | 78 | MCI/D A- | LBD | | | | 0,108 | 66 | 534 | 58 |
| **KSSG-47** | 73 | MCI/D A- | VDD | Posterior cortical atrophy | | 16 | 0,077 | 19 | | 58 |
| **KSSG-2** | 70 | MCI/D A+ | MCI AD | | | 24 | 0,041 | 138 | 850 | 55 |
| **KSSG-3** | 59 | MCI/D A+ | ADD | | Posterior Cortical Atrophy | 15 | 0,04 | 137 | 887 | 63 |
| **KSSG-4** | 78 | MCI/D A+ | Other | | Rapid progressive cognitive decline of unclear etiology | | 0,042 | 54 | 342 | 45 |
| **KSSG-6** | 56 | MCI/D A+ | ADD | | | 21 | 0,036 | 132 | 797 | 58 |
| **KSSG-11** | 70 | MCI/D A+ | ADD | | | 16 | 0,029 | 246 | 1575 | 68 |
| **KSSG-15** | 53 | MCI/D A+ | ADD | | | 20 | 0,043 | 76 | 545 | 75 |
| **KSSG-16** | 68 | MCI/D A+ | ADD | | | 19 | 0,044 | 114 | 666 | 65 |
| **KSSG-18** | 76 | MCI/D A+ | ADD | | | 15 | 0,034 | 156 | 957 | 85 |
| **KSSG-21** | 67 | MCI/D A+ | ADD | | Alcohol dependence | 18 | 0,042 | 83 | 495 | 86 |
| **KSSG-23** | 84 | MCI/D A+ | VDD | | Traumatic brain injury | 19 | 0,045 | 109 | 694 | 55 |
| **KSSG-25** | 70 | MCI/D A+ | ADD | Probable vascular dementia | | 12 | 0,032 | 110 | 781 | 45 |
| **KSSG-26** | 77 | MCI/D A+ | ADD | | | 16 | 0,04 | 101 | 576 | 68 |
| **KSSG-31** | 81 | MCI/D A+ | ADD | Probable vascular dementia | | 11 | 0,042 | 161 | 846 | 65 |
| **KSSG-36** | 73 | MCI/D A+ | ADD | | | 7 | 0,039 | 73 | 439 | 88 |
| **KSSG-38** | 90 | MCI/D A+ | ADD | | | 3 | 0,04 | 58 | 356 | 95 |
| **KSSG-39** | 65 | MCI/D A+ | MCI AD | | | 20 | 0,05 | 101 | 582 | 69 |
| **KSSG-40** | 84 | MCI/D A+ | ADD | | | 20 | 0,064 | 64 | 435 | 74 |
| **KSSG-48** | 57 | MCI/D A+ | ADD | | | 19 | 0,05 | 160 | 1077 | 65 |

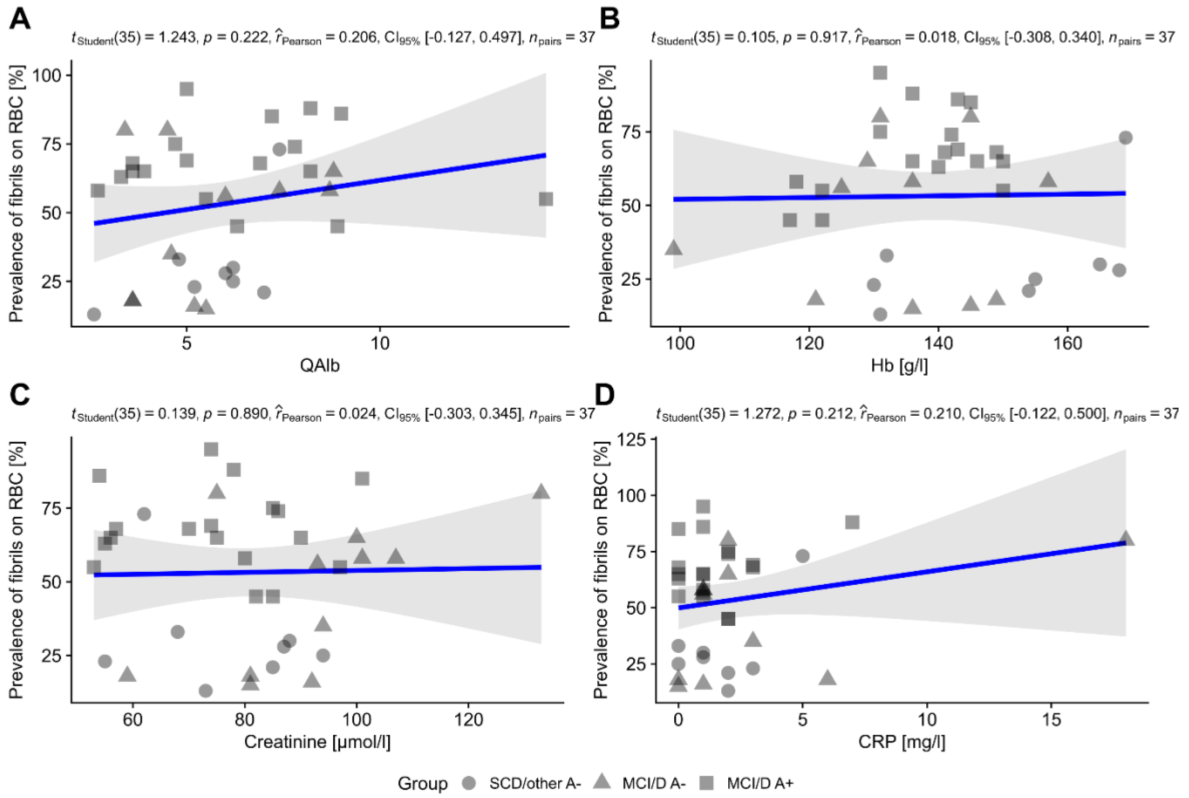


**Supplementary Figure 1**: Correlations between laboratory parameters and fibril prevalence on RBCs. (a) Correlation between the cerebrospinal fluid (CSF)/serum quotient of albumin (QAlb = $Albumin_{CSF}$ / $Albumin_{Serum}$ x1000) and the prevalence of fibrils on RBCs, (b) Correlation between hemoglobin concentration (Hb [g/l]) and the prevalence of fibrils on RBCs, (c) Correlation between creatinine levels (µmol/l) and the prevalence of fibrils on RBCs, (d) Correlation between C-reactive protein (CRP [mg/l]) levels and the prevalence of fibrils on RBCs. Note: A 85-year-old male patient with mild cognitive impairment (MCI) attributed to vascular disease presented with an elevated C-reactive protein (CRP) level of 18 mg/l. The etiology of the CRP elevation was not established in the Memory Clinic evaluation, and there were no clinical indications of an infectious disease.In all subfigures, the data points represent individual subjects, color-coded by group: SCD/other A- (subjective cognitive decline/other, amyloid-negative), MCI/D A+ (mild cognitive impairment/dementia, amyloid-positive), and MCI/D A- (mild cognitive impairment/dementia, amyloid-negative). The solid blue line indicates the line of best fit. Student's t-test and Pearson correlation coefficient (r) values are provided for each analysis, along with the corresponding confidence intervals and number of observations (npairs).
